# Supplementary material for: Diversity of bacteriophages encoding Panton-Valentine leukocidin in temporally and geographically related Staphylococcus aureus
Source: PLoS One. 2020 Feb 10;15(2):e0228676. doi: 10.1371/journal.pone.0228676 (PMC7010278; doi:10.1371/journal.pone.0228676)
Supplement: Attachment — Submitted filename: Response to Reviewers.docx [file pone.0228676.s001.docx]

We thank the reviewers for their time spent reviewing the manuscript and appreciate their suggestions. The author agrees that some parts of the presentation are confusing and difficult to read and acknowledges both reviewers for requesting simplification of nomenclature. The following changes have been made:

- In the Materials and Methods section the bacteriophage/prophage nomenclature has been explained and the author respectfully requests permission to leave it unchanged. So far there has not been any standardization of nomenclature for PVL bacteriophage in *S. aureus*. Following discussions with colleagues, the authors felt it was important for this and future publications to apply a meaningful nomenclature that would show at a glance which bacteriophage/prophage they were, their geographical provenance and the genetic background of their lysogen. To make the manuscript easier to read, where the bacteriophage occur in groups in the same sentence full designation has been given to only the first bacteriophage, for the remainder the prefix in front of the hyphen has been removed and only the bacteriophage -st designations separated by commas have been presented. Furthermore, to reduce the number of prophages mentioned, where the φSa2USA/φSa2wa-st93 group of bacteriophages had the same properties, the individual bacteriophages have been removed and the group designation inserted.
- Clone designations have been shortened by presenting the strain identity number as a subscript.
- Clone genotypes have been shortened by removing “MRSA” and the bracketed SCC*mec* descriptors from the designations.
- In addition to the changes requested by the reviewers the author feels that the second paragraph in the PVL gene sequencing section was overly complicated and difficult to comprehend. For several years now scientists have been sequencing the PVL genes and most publications have associated different alleles with the genetic lineage of the lysogen, however, this study shows that this is not the case, therefore we feel compelled to report the findings in detail. No information has changed. The main points have been numbered and sentences have been re-arranged to make the paragraph more comprehensible.

**Reviewer #1**

*This is a highly detailed genomic analysis of recombinational interactions among PVL-encoding prophages in Western Australia (WA), including data indicating that recently imported S. aureus strains with PVL-encoding prophages contributed to the remarkable recent increase in PVL prevalence in WA.*

The study was performed due to concern that the PVL bacteriophages from international MRSA clones were moving into the predominant local clones. We did not mean to imply that the international clones were responsible for the high rate of PVL-positive MRSA in WA. To address this issue, we have modified the text as follows:

- In the Abstract, a sentence has been added. “There was concern that PVL bacteriophages from the international clones were transferring into the local clones, therefore a comparative study of PVL-carrying φSa2 prophage genomes from historic WA PVL-positive *S. aureus* and representatives of all PVL-positive CA-MRSA isolated in WA between 2005 and 2008 was performed.”
- In the Introduction: Sentence change. “The rise in the number of PVL-positive CA-MRSA in WA since the first was found in 2003 has been alarming.”

*Given that the phenomenon of recombinational promiscuity among phages is very well known and has been thoroughly documented years ago, this paper represents merely a catalog of this phenomenon as applied to a particular set of prophages.*

- What this study is trying to do is further the scientific understanding of bacteriophage recombination and heterogeneity by using the Alpha aligner to identify modules and investigate at the sequence level exactly where the greatest diversity is, what form it takes and which modules are being exchanged.
- We acknowledge the known high rate of recombination by bacteriophages and in the Introduction extra information has been added as follows:

“It has been well documented that bacteriophages undergo high rates of recombination and both these forms of transmission allow opportunity for genetic exchange, the potential mechanisms being transposition, site-specific recombination, homing endonucleases and homologous and illegitimate recombination (11).”

**Minor points**

*1. Please explain how these strains were introduced into Australia.*

- In the Introduction: New sentence. “In WA, all MRSA are submitted to a central facility for typing and epidemiological investigation (21) and between 2005 and 2008 eight international PVL-positive CA-MRSA were introduced into WA.”

*2. It is implied, line 236, that φSa2wa-st78 used a different att site, but the data in Fig. 1 do not* *support this – please clarify.*

- There is no *att*Li for φSa2wa-st78 in Fig 2. It could not be identified.
- We have commented on this in the following unchanged sentence:

“ *att*Li of φSa2wa-st78 could not be identified, however its *att*Ri was reasonably similar (3 bp difference) to the φSa2wa prophages over the LH arm and the first 17 bp of the common core while 32 of the remaining 37 bp were different (Fig 2)”

- In the last paragraph of this section the difference is further discussed.

*Were any matching sequences between otherwise unrelated genes encountered, that could account* *for recombination, other than that mentioned in line 276?*

- There were matching sequences throughout however, given the plethora of mechanisms of recombination employed by bacteriophages we would not dare to speculate on how any recombinations may have occurred unless we could show evidence of the event (such as flanking repeats).

*4. Could the authors show (tabulate) which imported PVL phages lysogenized native strains intact, and which segments of these were incorporated by recombination into native prophages?*

- A Table would be too small. Only one PVL-positive native WA clone had a bacteriophage that was identical (97-100% homology) to one of the imported bacteriophages. That was WA5_18751_ lysogenised with φSa2wa-st8 which has been incorporated into the φSa2USA/φSa2wa-st93 group.
- Given the heterogeneity of the bacteriophages, the vastness of the geographical region and community they came from, and how very little is understood about bacteriophage recombination it would not be possible from this study to reliably predict where individual segments originated.

**Reviewer #2**

*“The genomes of PVL bacteriophages … have revealed an unexpected amount of diversity that has made it difficult to trace their origins.”*

- With the exception of the φSa2USA/φSa2wa-st93 group the authors were unable to get any bootstrap support for any phylogenetic analysis on this collection of PVL bacteriophage. This is remarkable in itself. The authors feel that the PVL prophage may be providing the lysogens with fitness properties other enhanced virulence from PVL production. This is why this study has looked so closely at the chromosomal attachment region and searched for novel or unique bacteriophage genes and modules. This will be for future studies.

*The prophages were sorted into different genera depending on “morphogenesis and structural genes.” What were these criteria? There are genera in this section that are mentioned but not fully described, i.e, the “3alikevirus” genus. To me, this doesn’t mean anything without a further definition of this genus.*

- The most recent reference on staphylococcal bacteriophage classification has been used. The classifications are based on the criteria therein. To direct readers to the references more clearly we have re-written a sentence as follows:

“The prophage genomes had the organisation of *Siphovirida*e family Sfi21-like PVL viruses of the *Caudovirales* order and, according to the most recent staphylococcal bacteriophage classification criteria, were placed into two genera and three PVL bacteriophage groups (Table 2) (34-36).”

- The most striking difference between the genera is that the 77likevirus genus consists of icosahedral-headed bacteriophage and the 3alikevirus genus are prolate-headed bacteriophage. This has been mentioned in the text and reinforced throughout the document.

*Titles like “sequence analysis, PVL gene sequencing, alpha alignments etc.” do not provide readers with any information on conclusions drawn.*

- The Results headings have been changed as requested.

*The results section “Induction and lysogenisation” (and corresponding Table 4) is confusing and lacking in detail. The authors should clearly state what was done, (i.e what strains were induced), which ones they were able to obtain bacteriophage from, why they used those strains, which recipient strains they attempted to infect …etc.*

- This section has been re-written to address the reviewers’ concerns. To keep consistency with the main focus of the study only induction experiments on the Australian clones have been reported and more details on what was done have been included. The author agrees that presenting the results of the international clones only added confusion. The Table has been re-formatted to include only the Australian clones with the control, and an extra column has been added to present the results of the different recipient/indicator strains.
- One sentence giving extra information has been added:

“The induced bacteriophages each lysogenised only one of the two indicator strains demonstrating some specificity of lysogenisation.”

*Data. The authors do not indicate if the bacteriophage sequences generated have been deposited in an online repository.*

- In the original submission, this data was under the heading “Sequence data availability” at the end of the Results section. It has now been moved to the first paragraph of the Results section to make it more prominent.

*Authors alternate the spelling of “defense” and “defence” throughout the manuscript. Please adjust to make uniform.*

- This has been done.

*In vitro is not always italicized, one example is in line 421.*

- This has been done.

*Figure 4 is difficult to interpret. The colors on the key don’t always match the colors on the actual figure. Specifically, the purple color designating ϕSa2wa-st80 either doesn’t appear on the figure or is a totally different shade on the figure.*

- The colors have been adjusted.

*The figure legend could be expanded to help explain the color code.*

- The following sentence has been adjusted in the Figure 4 legend:

“φSa2wa-st5 ORFs are represented as arrows indicating the direction of transcription and coloured according to the PVL prophage or groups of PVL prophages from PVL-positive *S. aureus* in WA that share 97 to 100% nucleotide identity.”

*It would also be useful to indicate the areas shown in Fig 1 and 2.*

- The Figure 1 (now Fig 2) sequences are attachment sites that are on the chromosome, proximal to the terminals of the prophages. This is explained in the text. “Chromosomal sequences proximal to the prophage terminals encoded the hybrid *att*Li and *att*Ri sites of the *att*B and *att*P sites on the chromosome and a circularly permuted form of the bacteriophage.” Because the prophage sequences were extracted from whole genomes it was not possible to determine which hybrid *att* site belonged to the bacteriophage and which was on the chromosome.
- The Figure 2 (Now Fig 3) region is in a part of Figure 1 that already is “cluttered” with information. The relevant ORFs are mentioned in the text and marked on Fig 1. To guide readers to the region in the text we have inserted a reference to Fig 1 as follows:

“The intergenic region between the divergently transcribed integrase gene, *int*, and its associated HP ORF, originally called *orfC* (39) (Fig 1), contained structures indicative of involvement in regulation and lysogeny in all prophages (Fig 3).”

*Fourteen prophages…(Table 1)”. Why does Table 1 have 17 phages listed?*

- Table 1 in the manuscript submitted has 14 prophages listed. The resubmitted manuscript has Table 1 with 14 prophages.

*Line 278: (Figure 4). Figures are out of order*

- The Figures have been re-numbered and re-inserted in the correct order

*Line 262 to 275. Overall the writing in this section was unclear and I was unable to follow the impact of Sa2wa-st772*

- This is the first description of the secondary genetic features which are predicted to be involved in lysogeny and regulation in φSa2 bacteriophages. The authors respectfully feel that the text, although somewhat complicated is supported by the figure and provides a guide for further investigations.
- The impact of the differences in φSa2wa-st772 have been addressed in a new paragraph in this section.
