# Supplementary material for: Diversity of bacteriophages encoding Panton-Valentine leukocidin in temporally and geographically related Staphylococcus aureus
Source: PLoS One. 2020 Feb 10;15(2):e0228676. doi: 10.1371/journal.pone.0228676 (PMC7010278; doi:10.1371/journal.pone.0228676)
Supplement: Attachment — Submitted filename: Response to reviewers.docx [file pone.0228676.s002.docx]

*Reviewer #2: My major concern with the previous version of the manuscript has not been addressed. While the authors present an extremely detailed analysis of 14 bacteriophage genomes there doesn't appear to be any solid conclusion or impact from the study. The manuscript has been modified to make it clearer and easier to understand but the overall impact of the study remains unclear.*

PVL-positive CA-MRSA have evolved to become one of the most significant bacterial pathogens in global communities. From an initial concern that well adapted local clones of PVL-negative CA-MRSA were acquiring the PVL-carrying bacteriophage from imported international CA-MRSA and consequently, the implications for morbidity and therapeutic management of infections in WA, the authors have told a story of the PVL-carrying bacteriophage in a defined geographical region from a point in time when there were no PVL-positive CA-MRSA to a situation where 52.8% of all CA-MRSA in WA were PVL positive. So far this is the only study that has addressed the epidemiology and genetics of a temporally and geographically related virulence-carrying staphylococcal bacteriophage.

Heterogeneity amongst bacteriophages is acknowledged and for the majority of bacteriophages in this study this is what was ultimately revealed however, one bacteriophage was shown to be remarkably stable over at least 20 years in different genetic backgrounds. In a time of the emergence of multiple antibiotic resistance in *S. aureus*, when several groups are investigating the utility of bacteriophages as therapeutic agents, this finding is important. If bacteriophages are to be used as therapeutic agents then their epidemiology and basic biology, such as regulation of lysogeny, need to be understood. Why is this bacteriophage so stable and successful in particularly virulent CA-MRSA? This study has used the Alpha Aligner to identify unique genes that may help answer this question. Furthermore, use of the Alpha Aligner has added to the understanding of basic bacteriophage biology by enabling a detailed analysis of the genetics of the most diverse region of the bacteriophages.

There is a paucity of information on the molecular biology of staphylococcal bacteriophages and the author respectively feels that the overall impact of the manuscript is that it that it presents new knowledge that can be used for further investigations into bacteriophage biology and genetics in CA-MRSA.

Changes to the manuscript

- The Abstract has been modified. The following statement has been removed because the authors did not feel it was relevant to the abstract:

“…however the role played by the toxin and the bacteriophage in *S. aureus* virulence has been controversial.”

- In the abstract the clones that probably acquired the PVL bacteriophage in the WA community have been named and reference to the fact that they did not disseminate has been included.
- To enable clearer assessment of the impact of the manuscript the focus of the study has been made clearer in the Introduction by numbering and a more logical reversing of the two aims. This is followed through in the Abstract.
- Throughout the manuscript reference to the chromosomal attachment site as the “attachment site” has been changed to “integration site”. This is to avoid confusion with the cell wall bacteriophage attachment site that is used in many publications.
- A comma has been deleted and another inserted to improve grammar and readability of the following sentence. “Overall, only two of the five indicator strains, RN4220 and WBG286 were lysogenised and specifically, only one in each induction experiment, demonstrating some specificity of lysogenisation (Table 4).”
- In the Discussion. In the first sentence the word “prophages” has replaced “bacteriophages”.
- In reference 44. The Journal has been correctly abbreviated.
